# Mice with a naturally occurring DISC1 mutation display a broad spectrum of behaviors associated to psychiatric disorders

**DOI:** 10.3389/fnbeh.2014.00253

**Published:** 2014-07-30

**Authors:** Raquel Gómez-Sintes, Mirna Kvajo, Joseph A. Gogos, José J. Lucas

**Affiliations:** ^1^Centro de Biología Molecular “Severo Ochoa”, (CBMSO) CSIC/UAMMadrid, Spain; ^2^Networking Research Center on Neurodegenerative Diseases (CIBERNED), Instituto de Salud Carlos IIIMadrid, Spain; ^3^Department of Psychiatry and Department of Physiology and Cellular Biophysics, Columbia University Medical CenterNew York, NY, USA

**Keywords:** DISC1, mouse model, 129 strain, pre-pulse inhibition, forced swim test, tail suspension test

## Abstract

Disrupted in schizophrenia-1 (DISC1) gene is associated with several neuropsychiatric disorders as it is disrupted by a balanced translocation involving chromosomes 1 and 11 in a large Scottish pedigree with high prevalence of schizophrenia, bipolar disorder and major depression. Since its identification, several mouse models with DISC1 genetic modifications have been generated using different approaches. Interestingly, a natural deletion of 25bp in the 129 mouse strain alters the DISC1 gene reading frame leading to a premature stop codon very close to the gene breakpoint in the mutant allele of the Scottish family. In the present study we confirmed that the 129DISC1^Del^ mutation results in reduced level of full length DISC1 in hippocampus of heterozygous mice and we have characterized the behavioral consequences of heterozygous 129DISC1^Del^ mutation in a mixed B6129 genetic background. We found alterations in spontaneous locomotor activity (hyperactivity in males and hypoactivity in females), deficits in pre-pulse inhibition (PPI) and also increased despair behavior in heterozygous 129DISC1^Del^ mice, thus reproducing typical behaviors associated to psychiatric disorders. Since this mouse strain is widely and commercially available, we propose it as an amenable tool to study DISC1-related biochemical alterations and psychiatric behaviors.

## Introduction

Among the many proposed susceptibility genes for schizophrenia, Disrupted in schizophrenia-1 (DISC1) is currently one of the best supported candidates (Arguello and Gogos, 2006; Ross et al., 2006). DISC1 was originally identified in a large Scottish family with a high prevalence of psychopathologies (Millar et al., 2000). In this family DISC1 expression is disrupted by a balanced translocation involving chromosomes 1 and 11. Although the name of DISC1 refers only to schizophrenia, carriers of the translocation in the Scottish family were diagnosed for several mental disorders including schizophrenia, bipolar disorder (BD) and major depression (MD; Blackwood et al., 2001; Wexler and Geschwind, 2011). Common variations in DISC1 may also play a role in schizophrenia and affective disorders in karyotypically normal patient populations (Gogos and Gerber, 2006). The role of DISC1 in neurodevelopment also supports the possibility of neurodevelopmental dysfunction in schizophrenic patients with DISC1 alterations (Arguello and Gogos, 2006; Ross et al., 2006).

As mentioned, the mental disorders reported in the Scottish family as a consequence of the DISC1 alteration include schizophrenia, BD and MD. Schizophrenia is a psychiatric disease affecting 1% of population worldwide. Patients are affected by positive symptoms (hallucinations, delusions, hyperactivity and thought disorder), negative symptoms (diminished social behavior, apathy, loss of energy and interests) and cognitive symptoms (deficit in working memory, poor executive functioning, trouble paying attention) (Ross et al., 2006). BD, also known as bipolar affective disorder or manic-depressive disorder, is characterized by recurrent episodes of mania (elated mood, hyperactivity) and depression (loss of interest, apathy, appetite loss, psychomotor retardation, suicidal thoughts) (Kato, 2008). BD and MD are commonly referred to as mood disorders (Jope and Roh, 2006).

When trying to generate a mouse model with a modified allele to express a truncated form of DISC1 with an interruption point close to the gene breakpoint in the Scottish family, Koike et al. (2006) discovered a natural *Disc1* mutation in the 129S6/SvEv mouse strain. This mutation termed 129DISC1^Del^ consist on a 25bp deletion in exon 6 that induces a frameshift in the reading frame resulting in 13 novel aminoacids and a premature stop codon in exon 7, thus abolishing production of full-length protein. In order to force the production of a truncated form, Koike et al. introduced a targeted mutation with an additional stop codon at exon 8 followed by a polyadenylation site. The combination of both the natural 129DISC1^Del^ mutation and the targeted one in B6 genetic background resulted in a deficit in working memory (Koike et al., 2006; Kvajo et al., 2008). Interestingly, the above mentioned naturally occurring 25bp deletion was reported to be common to all 129 mouse substrains (Clapcote and Roder, 2006).

In this study we aim to characterize the effect of the natural 129DISC1^Del^ mutation *per se* in a B6129F2 mixed background. Mice heterozygous for the 129DISC1^Del^ mutation showed alterations in spontaneous locomotor activity, as well as a deficit in pre-pulse inhibition (PPI), a test commonly used to study schizophrenia related-like behavior, and increased immobile time in the forced swim test (FST) and tail suspension test (TST), tests that measure behavioral despair. Together, these results suggest that easily available mice with 129DISC1^Del^ mutation can be used as a tool to model mental disorders.

## Materials and methods

### Animals

B6 and 129 mice were obtained from Jackson laboratories (C57BL/6J: stock number 000664 and 129S1/SvImJ: stock number 002448). All mice were housed at Centro de Biología Molecular “Severo Ochoa” animal facility. Mice were housed four per cage with food and water available *ad libitum* and maintained in a temperature-controlled environment at 22 ± 1°C on a 12/12 h light-dark cycle with light onset at 07:00 h. B6 and 129 mice were crossed to obtain B6129F1 hybrid mice that are heterozygous for the 129DISC1^Del^ mutation. B6 was chosen for being one of the most widely used mouse strains for behavioral studies. Experimental mice correspond to B6129F2 (from now on referred as B6129) generated from B6129F1 × B6129F1 crosses. Animal housing and maintenance protocols followed the guidelines of Council of Europe Convention ETS123, recently revised as indicated in the Directive 86/609/EEC. Animal experiments were performed under protocols (P15/P16/P18/P22) approved by the Centro de Biología Molecular Severo Ochoa Institutional Animal Care and Utilization Committee (Comité de Ética de Experimentación Animal del CBM, CEEA-CBM), Madrid, Spain.

### PCR

Each 50-μl PCR contains 18.75 μl nuclease-free water, 10 μl of 5× PCR buffer, 2.5 mM MgCl_2_, 0.2 mM of dNTPs (Promega), 2 mM of each primer (Sigma), 1.25 units of Taq DNA polymerase (Promega) and 200 ng genomic DNA. Reactions are incubated at 94°C for 5 min, followed by 36 cycles of 94°C for 1 min, 60°C for 1 min and 68°C for 1 min, followed by 68°C for 7 min. Following thermocycling, 10 μl of PCR product were electrophoresed at 90–100 V through a 3% (w/v) agarose gel in 1× TAE buffer. For discriminating the 25-bp deletion we performed PCR by using the following primers: TAG CCA CTC TCA TTG TCA GC (forward) and CTC CAT CCC TTC CAC TCA GC (reverse), that yielded a 179bp band from the wild type allele and a 196bp band from the mutant allele.

### Western blot analysis

Brains were quickly dissected on an ice-cold plate. Whole extracts from hippocampus (Hipp) and prefrontal cortex (PFC) (P14 mice) or dentate gyrus (DG), Cornu Ammonis (CA) and PFC (adult mice, 12 week-old mice) were prepared by homogenizing the brain areas in ice-cold extraction buffer consisting of 20 mM HEPES pH 7.4, 100 mM NaCl, 20 mM NaF, 1% Triton X-100, 1 mM sodium orthovanadate, 1 μM okadaic acid, 5 mM sodium pyrophosphate, 30 mM β-glycerophosphate, 5 mM EDTA, and protease inhibitors (2 mM PMSF, 10 μg/ml aprotinin, 10 μg/ml leupeptin and 10 μg/ml pepstatin). Samples were homogenized and centrifuged at 15,000 g for 20 min at 4°C. The resulting supernatant was collected, and protein content determined by Bradford assay. Fifteen micrograms (P14) or twenty-five micrograms (adult) of total protein were electrophoresed on 10% SDS-polyacrylamide gel and transferred to a nitrocellulose membrane (Schleicher and Schuell). The membranes were incubated with primary antibodies, polyclonal anti-N-terminal-DISC1 (1:100, generated in Dr. Joseph Gogos’ lab) or monoclonal anti-β-actin (1:5000, Sigma), overnight at 4°C in 5% non-fat dried milk in 0.1% TBS-Tween 20. Secondary antibodies used were polyclonal goat anti-rabbit immunoglobulins/HRP or polyclonal rabbit anti-mouse immunoglobulins/HRP (DAKO Cytomation) (1:2000) and enhanced chemilumescent (ECL) detection reagents (Perkin Elmer) were used for immunodetection. The number of animals used for biochemical analysis was: +/+ *n* = 6, +/del *n* = 4, del/del *n* = 4 P14 mice and +/+ *n* = 4, +/del *n* = 6, del/del *n* = 4 12 week-old mice (adult).

### Behavioral testing

Behavioral test were conducted on sex and genotype balanced groups of 20–30 mice at 12 weeks of age. No effects of sex or sex*genotype were detected by two-way ANOVA in behavioral test except for open field, so data were presented for males and females separately and also data combining both sexes were analyzed and shown when statistically comparable. All behavioral studies were performed during light phase. Three or four independent groups of mice were tested separately and data were pooled together. Sequence of behavioral tests was: day 1, open field; day 2, habituation to startle/PPI apparatus (morning) and startle test (afternoon); day 3, PPI test; day 6, fear conditioning training; day 7, contextual fear conditioning test (morning) and tone dependent test (afternoon); day 8; FST. Two different groups of animals performed TST followed by spatial memory (Y-maze). The number of animals used for each individual behavioral test is detailed in the figure legends.

#### Open field

Locomotor activity was measured in clear plexiglas boxes measuring 43.2 cm × 43.2 cm, outfitted with photo-beam detectors for monitoring horizontal and vertical activity. Activity levels were recorded with a MED Associates’ Activity Monitor (MED Associates, St. Albans, VT). Locomotor activity data were collected via a PC and were analyzed with the MED Associates’ Activity Monitor Data Analysis software. Mice were placed in a corner of the open-field apparatus and left to move freely. Variables recorded included: resting time (s), ambulatory time (s), vertical/rearing time (s), jump time (s), stereotypic time (s) and average velocity (cm/s). Mice were not exposed to the chamber before testing. Data were individually recorded for each animal during 30 min.

#### Startle response

Startle response curve as well as PPI test were conducted using a commercially available StartFear (Panlab-Harvard Apparatus). The StartFear Combined system is a polyvalent system for conducting both startle reflex/PPI and fear conditioning experiments in one same enclosure. This system allows recording and analysis of the signal generated by the animal movement through a high sensitivity weight transducer system. Protocol was adapted from Stark et al. (2008). Briefly, a startle response curve session was performed the day before PPI in order to rule out any impairment in hearing. Startle measures included recordings made every 4 dBs above background (66 dB), up to 118 dB. We placed each mouse in the chamber and gave it a 5-min acclimation period, during which background white noise was continually present. Each mouse received four times each trial type (40 ms-sound pulses from 70 dB to 118 dB) distributed randomly and separated by 10 s-intertrial interval. Response amplitude was considered as the maximum response level recorded during 1 s after the sound pulse.

#### Pre-pulse inhibition

Percent PPI was calculated as 100-[(startle response of acoustic startle from acoustic prepulse and startle stimulus trials/startle response alone trials) × 100]. Trial types, trial type presentation, and background noise levels were performed according to the protocols described previously (Mukai et al., 2004; Stark et al., 2008) with some modifications. After 5 min habituation period (66 dB white noise background), eight sets of four different trials distributed randomly with a variable intertrial time (10, 15 or 20 s) were presented to each mouse: trial 1, 40-ms, 120-dB noise burst alone; trials 2 and 3, 120-dB startle stimulus preceded 100 ms earlier by a 20-ms, 70, 74, 78 or 82-dB noise burst (pre-pulse); trial 4, no stimulus, background noise alone (66 dB). As for startle test, response amplitude was considered as the maximum response level recorded during 1 s after the sound pulse.

#### Forced swim test

Mice were individually placed in a 22 cm diameter white plastic cylinder filled to 11 cm with water at room temperature (22 ± 1°C) (tanks were filled with tap water the day before to temper). Four mice at a time were videotaped with a camera placed above the cylinders. The mouse was considered immobile when it was motionless or executing the minimal activity to keep afloat (tail movement was used as criterion). Immobility time was measured every minute and presented as percentage of time immobile per minute. Also the last 5 min of a 6 min trial were quantified as total immobile time and the time to the first 3-s immobility episode as latency to immobility. Evaluation was performed by a person blind to sex or genotype.

#### Tail suspension test

Mice were suspended by the tail in a gray plastic apparatus consisting on four independent compartments (54 cm height × 15 cm width × 11 cm depth) with a metallic bar on the top. The tail was introduced in a plastic cylinder of 4 cm length in order to avoid mice to climb by using their tails. Mice were hanged by the tail using an adhesive tape placed 2 cm apart from the bottom. The percentage of time spent immobile per minute was measured. All analysis were performed blind.

#### Fear conditioning

Fear conditioning was conducted using StarFear combined system (Panlab-Harvard Apparatus). We measured the percentage of time immobile. Immobility episode is defined as a complete lack of movement besides respiration for at least 1.5 s. Protocol was adapted from Paterlini et al. (2005). Conditioning was carried out in a chamber with a stainless steel grid floor in a sound attenuation box. The grid floor was wired to a shock generator and an auditory signal was supplied from a loudspeaker on the wall of the chamber. On the conditioning day, mice were individually placed into the conditioning chamber. After one 3-min exploratory period, each mouse was exposed to three tone-footshock pairings (tone (CS), 30 s; footshock, 2 s, 0.2 mA at the termination of the tone; separated by 1 min intertrial interval). One min after the third footshock, the mouse was returned to its home cage. In this session, chamber walls were black, any noise, light or fan were turned off, mice were placed directly on the grid floor and ethanol 70% was used to clean between animals. The following day (test day), contextual test for conditioned fear response was conducted in the morning. Fear conditioning to the context of the training chamber was assessed by returning each mouse to the same conditioning chamber and measuring immobility as fear behavior for 6 min with no tone or footshock delivered. In the afternoon of that test day, the context and handling of the mice were changed to assess conditioned fear of the tone alone: lemon extract was used to scent the chambers, the flooring and walls of the chambers were replaced with white plastic coverings, the ventilation fan and chamber light were turned on, a background noise was set at 67 dB, CR-36 mural disinfectant was used to clean between animals and a different experimenter carried out the test. Mice were placed in the chambers for 6 min. Immobility was assessed for a 3 min baseline period (pre-CS) followed by another 3 min for the conditioning tone (CS) during which the tone was presented persistently for 3 min. Immobility behavior was analyzed using StarFear software provided with the apparatus setting minimum time to register immobility episode to 1500 ms and threshold to 10–90.

#### Y-maze

In order to measure spatial memory, a Y-maze made of black plastic was used. Two different protocols were used 1 week apart. First, we assessed a spontaneous alternation test in which mice were placed in one of the arms (start arm) and left to move freely for 5 min. Alternation is defined as successive entries into the three arms on overlapping triplet sets and was calculated as the ratio of overlapping triplet sets/possible alternations (total number of arm entries minus 2) multiplied by 100. One week after, spatial recognition memory was analyzed. In this task, mice were trained in the Y-maze with one of the arms closed. The mouse was allowed to freely explore for 5 min. After exploration, the mouse was removed and placed back to the cage (training phase). One hour later, the originally blocked arm was opened and defined as the “novel arm”. Mice were placed in the Y-maze in the start arm and left to move freely. Percentage of entries in the novel arm was measured.

#### Statistical analysis

Statistical analysis was performed with SPSS 21.0. Data are presented as mean values ± S.E.M. The normality of the data was analyzed by Shapiro-Wilk test, followed by Levene test to determine if variances were homogeneous. DISC1 protein levels by Western blot were analyzed with Student *t*-test. The effects of 129DISC1^Del^ mutant mice on mouse behaviors were evaluated in male and female mice separately or pooled together. Behavioral effects were analyzed by performing a mixed model of two-way ANOVA with repeated measures, with the genotype and sex as independent variables (between-subjects factor) and the measure corresponding to a specific test as dependent variable (within-subjects factor). First, any effect of sex or sex*other factors interaction was evaluated. Then, statistically significant differences between subjects were determined for each particular behavioral test. Significant effects at individual time point or conditions (i.e., pre-pulse intensity or preCS/CS) were further explored with lower level ANOVAs. A critical value for significance of *p* < 0.05 was used throughout the study.

## Results

### Mutant 129DISC1^Del^ mice show decreased levels of full-length DISC1

**Figure 1:**
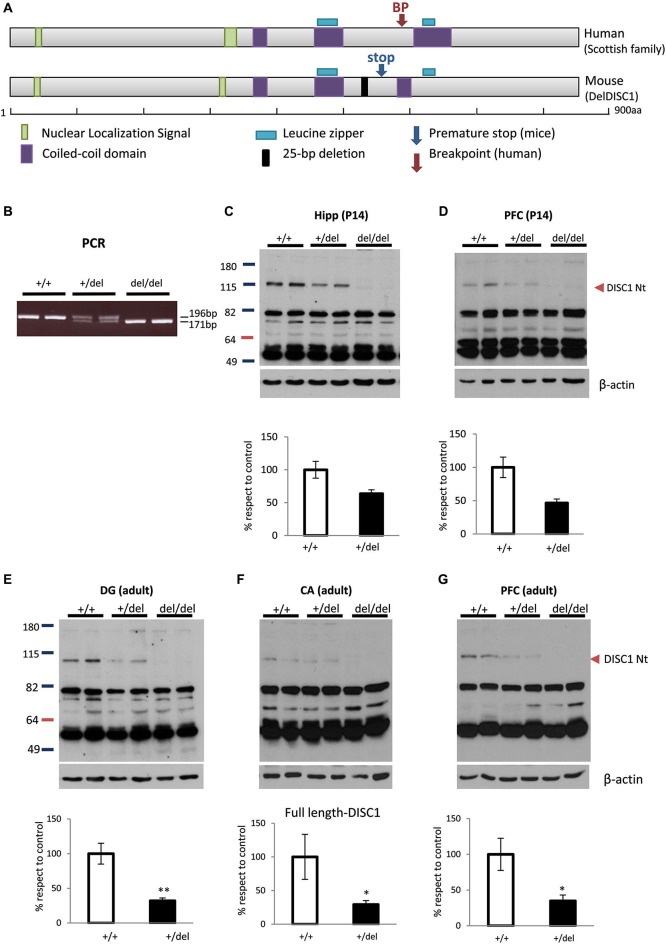
**Deficiency of full-length DISC1 in 129DISC1^Del^ mice.** **(A)** Scheme showing human DISC1 protein with breakpoint site in the Scottish family (upper) and mouse DISC1 protein with premature stop codon induced by 25bp deletion in 129 strains (lower). Both disruption points are very close and precede the c-terminal leucine zipper and coiled coil domains in both species. **(B)** Detection by PCR of a shorter band (176bp instead of 191bp) for mutant *disc1* gene in +/del and del/del mice. **(C–G)** Detection by Western blot and quantification of decreased levels (+/del) or absence (del/del) of full length DISC1 in hippocampus (**(C)**, *p* = 0.058) or prefrontal cortex (PFC) (**(D)**, *p* = 0.069) of P14 mice (*n*_+/+_ = 6, *n*_+/*del*_ = 4, *n*_*del/del*_ = 4) and dentate gyrus **(E)**, cornu Amonnis **(F)** or PFC **(G)** of adult mice (*n*_+/+_ = 4, *n*_+/*del*_ = 6, *n*_*del/del*_ = 4). * *p* < 0.05, **p < 0.01.

Figure 1A shows the resemblance between the gene breakpoint in the Scottish DISC1 mutation and the premature stop codon in 129DISC1^Del^ mutant mice. The balanced translocation involving chromosomes 1 and 11 in the Scottish family introduces a breakpoint immediately after exon 8 in *DISC1* gene. In 129 mouse strains a 25-bp deletion in exon 6 induces a frameshift in the reading frame that introduces a premature stop codon in exon 7 of *disc1*. Although the interruption sites are not exactly the same in human and mice, they both precede the C-terminal leucine zipper and the coiled-coil domains (Figure 1A).

To study the biochemical and behavioral consequences of the natural 129DISC1^Del^ mutation, we generated wild type (+/+), heterozygous (+/del) and homozygous (del/del) mice for this mutation. More precisely, B6 and 129 mice were crossbred to generate B6129F1 hybrid mice that are heterozygous for the 129DISC1^Del^ mutation. Then, B6129F1 mice were bred to generate an F2 in which the three possible genotypes were distinguished by PCR with primers designed to hybridize regions flanking the 25-bp deletion (Clapcote and Roder, 2006). A 196bp band was present in +/+ and +/del mice and a 171bp band was present in +/del and del/del mice (Figure 1B).The three genotypes were obtained with frequencies according to the expected Mendelian distribution (data not shown). We then confirmed by Western blot with an N-terminal DISC1 antibody the absence and reduction of full length protein in hippocampus of del/del and +/del mice respectively. More precisely, we found a strong decrease in hippocampus (*p* = 0.058; Figure 1C) and PFC (*p* = 0.069; Figure 1D) of P14 +/del mice and also a decrease in DG (*p* < 0.005; Figure 1E), CA regions (*p* < 0.05; Figure 1F) and in PFC (*p* < 0.05; Figure 1G) of adult +/del mice compared to +/+ mice.

### Heterozygous 129DISC1^Del^ mutant mice grow normally but show alterations in general activity

**Figure 2:**
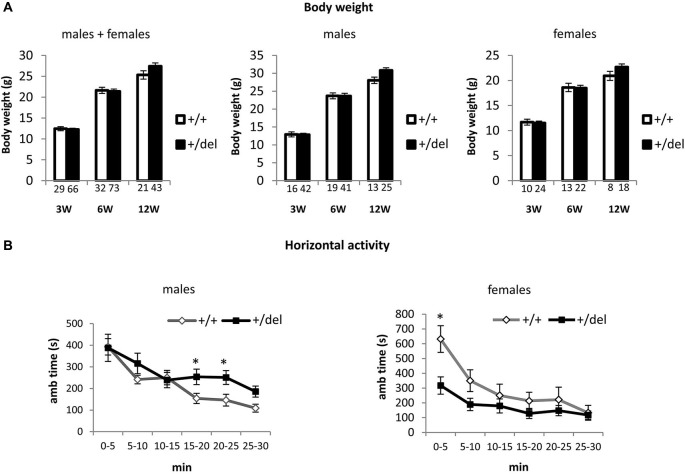
**Heterozygous mice grow normally but display alterations in spontaneous locomotor activity.** **(A)** Body weight was measured at the age of 3, 6 and 12 weeks and was represented by pooling together males and females (left panel), only males (central panel) and only females (right panel). Number of animals is detailed at the bottom of each bar in the graphs. **(B)** Horizontal activity was measured as ambulatory time in the open field over a 30 min trial divided in 5 min blocks. Males showed hyperactivity in the last part of the experimental time (left panel; *n*_+/+_ = 10, *n*_+/*del*_ = 12) by contrast females showed hypoactivity (right panel; *n*+/+ = 5, *n*+/del = 7). * *p* < 0.05.

Affected individuals in the Scottish pedigree are heterozygous for the DISC1 mutation. Accordingly, for the behavioral characterization of 129DISC1^Del^ mutant mice as a potential model to study DISC1 associated psychiatric behaviors, we focused our study on +/del mice as compared to +/+ mice. As a measure of general assessment, we first analyzed body weight at 3, 6 and 12 weeks of age and no differences were detected (Figure 2A). To analyze the consequences of decreased full-length DISC1 on behavior we started by assessing the general response to a novel environment and we tested the activity of 12 weeks old mice in the open field for 30 min. In the analysis, we first evaluated influence of sex and found that there was a statistically significant interaction between sex and genotype on ambulatory time (*F*_(1,31)_ = 7.50, *p* = 0.010), so data are presented in this case for males and females separately. We found increased horizontal activity in heterozygous (+/del) males compared to wt (+/+) littermates in the second half of the testing time, as shown by increased ambulatory time from minute 15 (Figure 2B, left panel). Two-way mixed ANOVA with repeated measures showed that there was a statistically significant effect of time (*F*_(3,63)_ = 4.99, *p* = 0.004) and interaction time*genotype on horizontal activity (*F*_(3,63)_ = 2.97, *p* = 0.04). By testing separate blocks of 5 min with one-way ANOVA, statistically significant differences in ambulatory time between genotypes were found at 15–20 min block (*F*_(1, 21)_ = 4.75, *p* = 0.041) and at 20–25 min block (*F*_(1,21)_ = 5.75, *p* = 0.026). On the contrary, heterozygous females (+/del) presented hypoactivity compared to wt (+/+) littermates in the first half of the testing time, thus showing an effect of decreased exploratory behavior in a novel environment. Two-way mixed ANOVA showed that there was a statistically significant effect of time (*F*_(1,10)_ = 33.13, *p* < 0.0005) and interaction time*genotype on horizontal activity (*F*_(1,10)_ = 4.64, *p* = 0.05). Separate blocks analysis by one-way ANOVA revealed significantly different ambulatory time in the first 5 min (*F*_(1,10)_ = 9.43, *p* = 0.012; Figure 2B, right panel).

### Decreased pre-pulse inhibition in heterozygous 129DISC1^Del^ mutant mice

**Figure 3:**
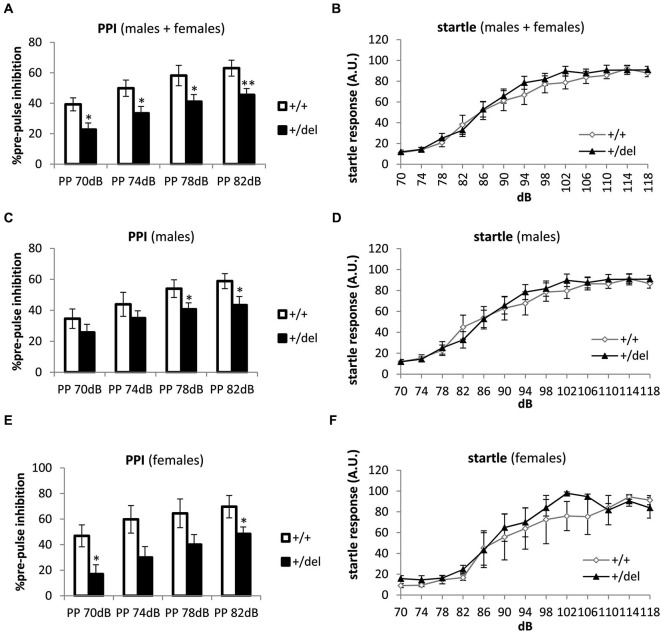
**Deficit in pre-pulse inhibition in heterozygous 129DISC1^Del^ mice.** **(A, C, E)** Percentage of PPI at 70, 74, 78 and 82 dB pre-pulses. Males and females (*n*_+/+_ = 15, *n*_+/*del*_ = 21 **(A)**); males (*n*_+/+_ = 10, *n*_+/*del*_ = 12 **(C)**); and females (*n*_+/+_ = 4, *n*_+/*del*_ = 8 **(E)**). **(B, D, F)** Startle response at different sound intensities: 70, 74, 78, 82, 86, 90, 94, 98, 102, 106, 110, 114 and 118 dB. Males and females **(B)**, males **(D)** and females **(F)**. * Correspond to analysis of each pre-pulse intensity separately (although two-way repeated measures ANOVA was also performed); * *p* < 0.05, **p < 0.01.

To determine whether specific behavioral deficits more related to schizophrenia and other psychiatric disorders are present in heterozygous (+/del) mice, PPI was performed. PPI test is a widely used test to assess sensorimotor gaiting, defined as the capacity of filtering incoming information, in animal models of schizophrenia and other mental diseases (Powell et al., 2009; Kohl et al., 2013). Results of this test are extensively accepted for evaluating schizophrenia-like behaviors due to the almost identically way of testing in mouse and humans (Arguello and Gogos, 2006). We designed the test with pre-pulses of 70, 74, 78 and 82 dB presented 20 ms before a 120 dB-pulse. Startle response was measured along the first second after the pulse. As a result of PPI test we found that heterozygous animals display decreased PPI compared to wt littermates when data of males and females were pooled together (Figure 3A). Previously, any effect of sex was determined by two-way ANOVA with repeated measures and no effect of sex (*F*_(1,31)_ = 0.643, *p* = 0.429) or interaction sex*genotype (*F*_(1,31)_ = 1.006, *p* = 0.324), sex*prepulse intensity (*F*_(3,93)_ = 0.476, *p* = 0.700) or sex*genotype*prepulse intensity (*F*_(3,93)_ = 1.211, *p* = 0.310) was found. Two-way repeated measures ANOVA showed a significant effect of prepulse intensity (*F*_(3,93)_ = 38.263, *p* < 0.0005) and of genotype (*F*_(1,31)_ = 9.426, *p* = 0.004) but not prepulse intensity*genotype interaction when males and females were pooled together and similar results were obtained by separating data from both sex. When analyzing results at different prepulses separately by one-way ANOVA, there were significant differences between genotypes at all pre-pulses assayed, PP70dB (*F*_(1,32)_ = 5.61, *p* = 0.024), PP74dB (*F*_(1,32)_ = 4.40, *p* = 0.044), PP78dB (*F*_(1,32)_ = 6.70, *p* = 0.014) and PP82dB (*F*_(1,32)_ = 8.24, *p* = 0.007) in males and females pooled together (Figure 3A). When data were split by sex, we found significantly decreased PPI in males at PP78dB (*F*_(1,20)_ = 4.40, *p* = 0.049) and at PP82dB (*F*_(1,20)_ = 4.89, *p* = 0.039; Figure 3C). In females, significant differences were found at PP70dB (*F*_(1,12)_ = 6.79, *p* = 0.024) and at PP82dB (*F*_(1,12)_ = 5.63, *p* = 0.037; Figure 3E). To discard interference of any potential hearing impairment we had previously assayed a startle response test. No differences were found when pooling together males and females (Figure 3B), nor when males (Figure 3D) and females (Figure 3F) were analyzed separately. Thus, these results show that heterozygous 129DISC1^Del^ mutant mice have deficits in sensorimotor gating in the PPI test.

### Heterozygous 129DISC1^Del^ mice spend more time immobile in the forced swim test and tail suspension test

**Figure 4:**
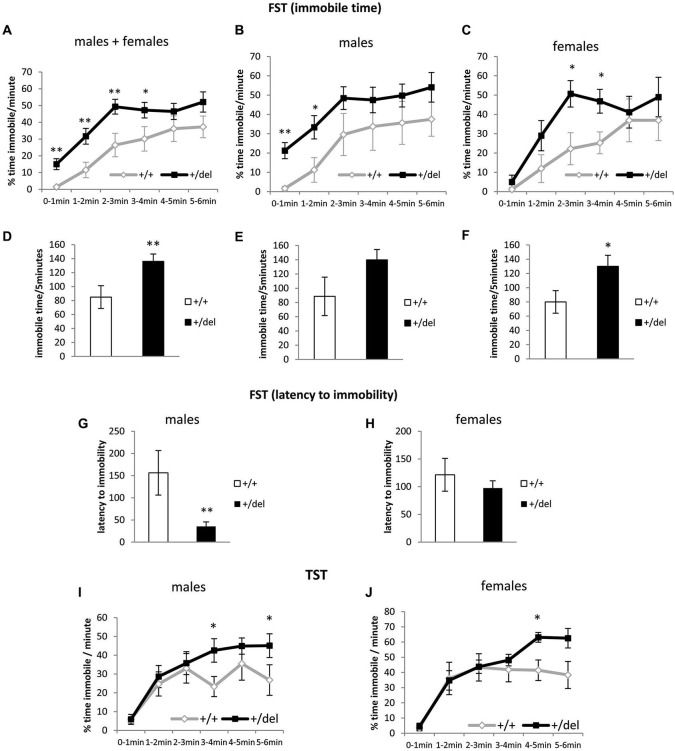
**Heterozygous 129DISC1^Del^ mice show increased immobility time in despair behavioral tests. (A–H)** Performance in forced swim test. **(A–C)** show percentage of time immobile per minute over the whole 6-min trial. **(D–F)** show total immobility time in the last 5 min of test. **(G–H)** show latency to the first immobility period (minimum 3 s). Males and females (*n*_+/+_ = 14, *n*_+/*del*_ = 26; left panels), males (*n*_+/+_ = 8, *n*_+/*del*_ = 16; central panels) and females (*n*_+/+_ = 6, *n*_+/*del*_ = 10; right panels). **(I–J)** Performance in TST represented as % of time immobile per minute over the whole 6-min trial. Males (**(I)**; *n*_+/+_ = 12, *n*_+/*del*_ = 15) and females (**(J)**; *n*_+/+_ = 8, *n*_+/*del*_ = 15).

We then performed the FST (Porsolt et al., 1978) and the TST (Castagné et al., 2011) to characterize +/del mice in terms of depressive behavior (Petit-Demouliere et al., 2005; Castagné et al., 2011). As shown in Figure 4, +/del mice spent more time immobile than their corresponding +/+ littermates (Figures 4A–F). Data of immobility time are presented as % time immobile per minute and also as total immobility time in the last 5 min of the test. When we analyzed % of time immobile per minute we found significant differences by two-way ANOVA of repeated measures in males and females pooled together (time: *F*_(4,144)_ = 7.631, *p* < 0.0005; genotype: *F*_(1,36)_ = 8.94, *p* = 0.013; no interaction time*genotype, Figure 4A) and similar results were obtained when both groups were separately analyzed (Figures 4B,C). One way-ANOVA was used to separately analyze time blocks and statistically significant differences were found between genotypes in the first (*F*_(1,38)_ = 9.064, *p* = 0.005), second (*F*_(1,38)_ = 7.699, *p* = 0.009), third (*F*_(1,38)_ = 8.414, *p* = 0.006) and fourth time blocks (*F*_(1,38)_ = 4.300, *p* = 0.045) for males and females analyzed together. Previously, we had evaluated potential sex differences by two-way ANOVA of repeated measures and found no effect of sex (*F*_(1,36)_ = 0.225, *p* = 0.638) or sex*genotype (*F*_(1,36)_ = 0.001, *p* = 0.975), sex*time (*F*_(4,144)_ = 0.031, *p* = 0.998) or sex*genotype*time (*F*_(4,144)_ = 0.460, *p* = 0.765) interaction. Furthermore, we found significant differences in total immobile time (Figures 4D–F) in males and females pooled together (*F*_(1,38)_ = 7.47, *p* = 0.009; one-way ANOVA) and females separately (*F*_(1,14)_ = 4.5, *p* = 0.05) but only a tendency was found in males (*F*_(1,22)_ = 3.38, *p* = 0.079). Moreover, evaluation of the latency to immobility has been suggested to increase the sensitivity of this test (Castagné et al., 2009b). In this case we found an effect of sex*genotype interaction (*F*_(1,36)_ = 4.097, *p* = 0.05). Accordingly, we found decreased latency to immobility only in +/del male mice (*F*_(1,22)_ = 10.32, *p* = 0.004; one-way ANOVA; Figures 4G,H). To further explore depressive behaviors, we also performed an experiment of TST, another behavioral despair test that complements FST avoiding confounding factor of water temperature and potential hypothermia (Castagné et al., 2011). In this analysis, an effect of sex (*F*_(1,48)_ = 8.713, *p* = 0.05) was found. Nevertheless, two-way ANOVA with repeated measures showed a significant effect of time (*F*_(5,125)_ = 24.741, *p* < 0.0005) and close to significant effect of time*genotype interaction (*F*_(5,125)_ = 24.741, *p* = 0.073) for males (Figure 4I) and a significant effect of time (*F*_(5,100)_ = 25.619, *p* < 0.0005) and close to significant effect of time*genotype interaction (*F*_(5,125)_ = 2.046, *p* = 0.079) for females (Figure 4J). One way-ANOVA was used to analyze minutes separately and statistically significant differences were found between genotypes in the fourth (*F*_(1,25)_ = 5.794, *p* = 0.024) and sixth minutes (*F*_(1,25)_ = 4.636, *p* = 0.047). Similar results were obtained when females were analyzed separately, but only the fifth minute reached statistical significance (*F*_(1,20)_ = 8.576, *p* = 0.008).

### Heterozygous 129DISC1^Del^ mice show normal performance in learning and memory tests

**Figure 5:**
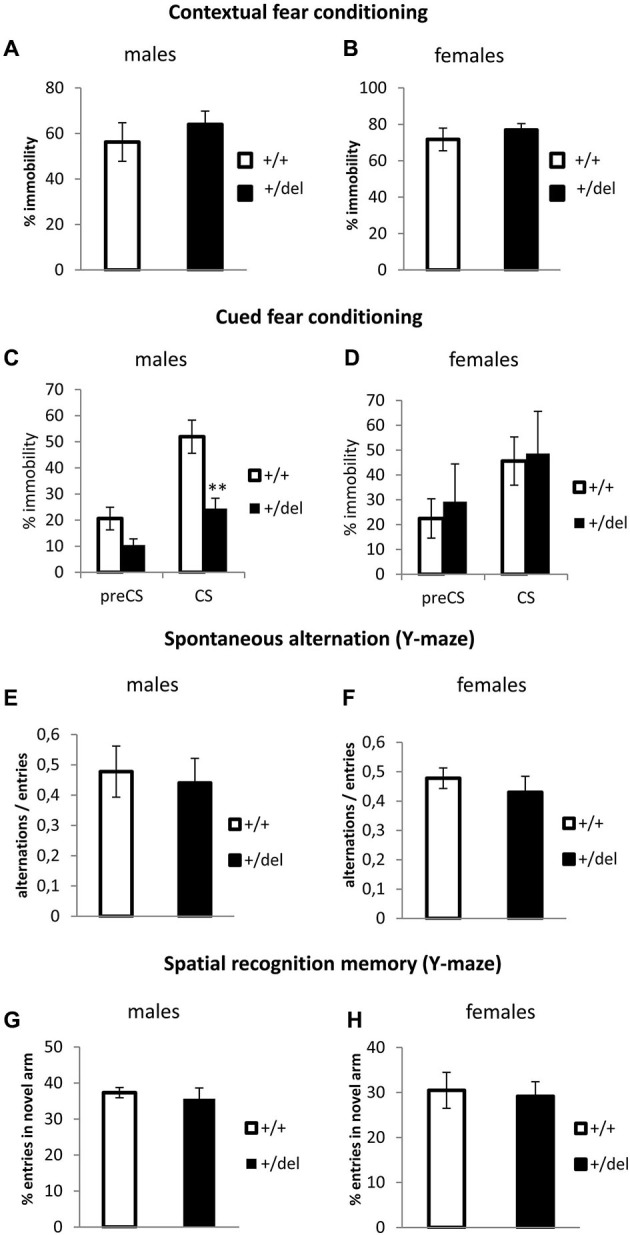
**Learning and memory test performance by heterozygous 129DISC1^Del^ mice.** **(A–B)** Contextual fear conditioning. Percentage of immobility during the 6 min trial on the second day of test. Males (**(A)**; *n*_+/+_ = 11, *n*_+/*del*_ = 15) and females (**(B)**; *n*_+/+_ = 6, *n*_+/*del*_ = 7, right panel). **(C–D)** Cued fear conditioning. Percentage of immobility during the 3 min before of the presentation of tone (preCS) and during the 3 min length of acoustic cue (CS). Males (**(C)**; *n*_+/+_ = 13, *n*_+/*del*_ = 11) and females (**(D)**; *n*_+/+_ = 10, *n*_+/*del*_ = 5). **(E–F)** Spontaneous alternation was measured in a Y-maze. Data are represented as the ratio between the number of overlapping triple alternate entries and total possible alternations in males **(E)** and females **(F)**. **(G–H)** Spatial recognition memory was measured in a Y-maze. Data are represented as the percentage of entries in the novel arm for males **(G)** and females **(H)**. Number of animals for **(E–H)** is: males (*n*_+/+_ = 6, *n*_+/*del*_ = 19) and females (*n*_+/+_ = 15, *n*_+/*del*_ = 19).

Together with attentional processes (classically detected by PPI), memory and learning deficits are considered cognitive symptoms of schizophrenia. In order to evaluate associative learning and memory in our model, we performed the fear conditioning test. In this test, mice will show a fear behavior, here quantified as immobility (absence of movement besides breathing) when they remember the context or a cue (tone) associated with an aversive stimulus (foot shock). No differences were found in the learning phase (training conditioning day) (data not shown) or in the contextual memory (Figures 5A,B). Interestingly when assaying cued test (Figures 5C,D), male heterozygous mice showed a decrease in the percentage of immobility during the conditioned stimulus phase (CS; Figure 5C), while heterozygous females showed no effect in cued fear conditioning (Figure 5D). Two-way ANOVA of repeated measures (preCS, CS) showed an effect of time (*F*_(1,22)_ = 45.92, *p* < 0.0005) and interaction time*genotype (*F*_(1,22)_ = 5.90, *p* = 0.024) for males. As we observed a decrease in time immobile in heterozygous males with respect to wt males both before and after the tone, we cannot ascertain if this represents a real deficit in cued fear conditioning or an effect of the novel context. In fact, when normalizing immobility during CS related to its corresponding preCS, percentages of change were comparable between wt and heterozygous males. An effect of sex was found by repeated measures two-way ANOVA (sex*genotype interaction: *F*_(1,31)_ = 0.221, *p* = 0.012). Spatial memory was also assessed in the Y-maze. First, we measured spontaneous alternation (Figures 5E,F), defined as successive entries into the three arms on overlapping triplet sets which is associated with short-term memory. Then, we studied spatial recognition memory as the percentage of entries in a novel arm that was blocked in a training trial (Figures 5G,H). We found no differences in any spatial memory test.

## Discussion

We have generated a new mouse model of DISC1 deficiency. Specifically, these mice have a heterozygous natural 25bp deletion inducing a premature stop codon and preventing formation of full-length DISC1 and thus mimic the effect on the DISC1 gene of the balanced translocation between chromosomes 1 and 11 occurring in the Scottish family. The here described heterozygous 129DISC1^Del^ mice show alterations in spontaneous locomotor activity, namely hyperactivity in males and hypoactivity in females, as well as deficits in PPI, a possible alteration in cued fear conditioning and also increased immobile time in the FST and TST.

Since the discovery of DISC1 as a susceptibility gene for mental disorders, several groups have directed their efforts into creating animal models for these diseases based upon DISC1 modifications *per se* (Arguello and Gogos, 2006; Jaaro-Peled, 2009; Pletnikov, 2009) or in combination with environmental factors such as stress (Cash-Padgett and Jaaro-Peled, 2013). One of such genetic mouse models is the gene targeted mouse line, *Disc1*^Tm1Kara^ that was generated and characterized by J. Gogos and coworkers and that we have already mentioned in the introduction (Koike et al., 2006; Kvajo et al., 2008, 2011). Besides, some transgenic models have been generated with expression of a truncated form of human DISC1 (ΔC or cc) that is believed to act as a dominant negative, two of them are constitutive: CaMKII-ΔC and BAC-ΔC (Hikida et al., 2007; Shen et al., 2008) and three are inducible: CaMKII-tTA-cc, CaMKII-tTA-ΔC and GFAP-tTA-ΔC (Li et al., 2007; Pletnikov et al., 2008; Ma et al., 2013). Moreover, mutant mice were created with point mutations in exon 2 of DISC1 by ENU-induced mutagenesis that resulted in two different models: Q41L and L100P (Clapcote et al., 2007). More recently, a mutant mouse lacking exons 2 and 3 (Δ2-3/Δ2-3) was generated (Kuroda et al., 2011). Regarding behavioral phenotypes, most of DISC1 mouse models show cognitive abnormalities in attention, demonstrated by alterations in PPI or latent inhibition, and impaired working memory (delayed non-match to place task, DNMTP). These cognitive abnormalities could be a good correlate of cognitive symptoms described for schizophrenia (Arguello and Gogos, 2006). In our study, the most robust finding in this direction is the impaired PPI that is observed in males and females together or separately. Regarding learning and memory, only Δ2-3/Δ2-3 mice (Kuroda et al., 2011) and ours showed alterations although in different tests. Kuroda et al. (2011) model showed impairment in contextual fear conditioning, a more hippocampal dependent task, and heterozygous 129DISC1^Del^ mice displayed a potential deficit in tone dependent fear conditioning, a task more sensitive to amygdalar alterations.

To model positive symptoms in schizophrenia or mania-related behaviors in BD, hyperactivity in response to stress or novelty and hypersensitivity to psycho stimulants have been used (Arguello and Gogos, 2006). Particularly, hyperactivity in a novel environment, as tested in an open field, could reflect psychomotor agitation (Jaaro-Peled, 2009). This characteristic behavior has been also shown by the L100P model and CaMKII-tTA-ΔC (Clapcote et al., 2007; Pletnikov et al., 2008). Particularly, in CaMKII-tTA-ΔC and in ours, it is an effect occurring only in males. On the contrary, +/del females present hypoactivity immediately after the transfer to the new environment possibly indicating an anxiety component (Gould and Dao, 2009) considered as negative symptoms in schizophrenia or depressive behaviors characteristic of BD and MD (Arguello and Gogos, 2006; Castagné et al., 2009a). This possible anxiety component should be further explored with more specific behavioral tests (Cryan and Holmes, 2005). Interestingly, GFAP-tTA-ΔC mouse model, expressing dominant negative DISC1 in astrocytes seems to behave similar to our model regarding spontaneous locomotion in open field. Although it was not studied in detail and further studies should be done, a slight trend to hyperactivity in males and hypoactivity in females can be observed before treatment with MK-801 (Li et al., 2007; Pletnikov et al., 2008; Ma et al., 2013). In humans, diagnosis of schizophrenia is 1.4 times more frequent in men and typically appears earlier (Picchioni and Murray, 2007). By contrast females have increased risk of depression subtypes as MD, dysthymia, atypical and seasonal depression but not for BD (Piccinelli and Wilkinson, 2000). The here observed results of increased activity in males and decreased activity in females might be possibly reflecting behaviors more related to schizophrenia in males and more related to depression in females. Also it is important to note differences in behavioral responses to a stressful situation as a novel environment that could be affecting distinct performance in the open field test (Goel and Bale, 2009).

Finally, FST is the most widely used assay to evaluate the validity of antidepressants. It reflects despair, a significant feature of MD and depressive episodes in BD, and that is also considered a negative symptom in schizophrenia (Arguello and Gogos, 2006; Castagné et al., 2009a). Similarly, TST is sensitive to despair behavior (Cryan et al., 2005). Increased immobility time in the FST was found in all models assayed except for L100P and Δ2-3/Δ2-3 (Clapcote et al., 2007; Kuroda et al., 2011). In BAC-ΔC model and ours (both maintained in a mixed background) the result in FST was validated with similar results in TST. TST presents the advantage of avoiding water temperature factor and potential hypothermia induction in the mice.

It is interesting to point that a single translocation-mutation produces different phenotypes in the Scottish family, clinically diagnosed as different but related psychiatric disorders (Brandon and Sawa, 2011). Thus, it is not surprising to observe variability within individuals of a specific model and phenotypes related to different psychiatric symptoms. Besides, the above mentioned differences when analyzing behavioral phenotypes in the various DISC1 mouse models are probably due to the differences in the specific DISC1 genetic modification and the strain background. The different animal models here mentioned have been generated using diverse approaches from targeted mutation strategies to dominant negative based models. As a result, different genetic scenarios vary from mutants, with one (heterozygous) or two (homozygous) dysfunctional copies of *disc1* gene, to animals with two intact copies plus an extra copy (dominant negative) encoding C-terminal or N-terminal part of the protein, which expression is driven by different promoters. Deficit or absence of full-length DISC1 protein or overexpression of a dominant negative form (N-terminal or C-terminal) of DISC1 can be altering the interaction of DISC1 protein with its numerous partner proteins depending on the region of the protein lacking, the time point of expression or the artificially stabilization of truncated forms/isoforms, thus modifying functions differently. Moreover, the different DISC1 models are maintained in different strain backgrounds which could affect differential results in behavioral analyses. Although a pure C57BL/6 is the most commonly used, some groups performed behavioral test in mixed backgrounds as BAC-ΔC, maintained in CBAB6 background (Shen et al., 2008); CaMKII-tTA-ΔC, maintained in a hybrid B6;SjL;CBA background (Pletnikov et al., 2008), and our 129DISC1^Del^ mice maintained in B6129 mixed background.

Focusing on the similarities with the Scottish pedigree, some of the mouse models are based on the generation of a truncated protein to mimic the potential truncated form in humans as CaMKII-ΔC, BAC-ΔC, CaMKII-tTA-ΔC and GFAP-tTA-ΔC or *Disc1*^*Tm*1*Kara*^ but the presence of the truncated protein has not been validated in human as translocation produces an mRNA that might not be stable because polyadenilation is probably missing (Kellendonk et al., 2009). Regarding the specific DISC1 genetic modification in the here described model, we would like to highlight that, among the mentioned mouse models, is the one that most closely mimics the human mutation in terms of having only two DISC1 alleles, one wild type and one mutated. Furthermore, our model presents the modification in the mutated allele most similar to the one in the Scottish pedigree as it introduces a breakpoint in equivalent position in the DISC1 gene. Furthermore, it is worth noting that the here described model is the only one that, apart from hyperactivity in a novel environment (males), also displays the combination of impaired PPI, possibly altered learning and memory and increased immobility time in the FST and TST that are paradigmatic mouse behaviors associated to schizophrenia and depression. In summary, 129DISC1^Del^ mutation renders the only mouse model that recapitulates behaviors reflecting all types of symptoms: positive, negative and cognitive behaviors associated to DISC1 alterations.

Recently, the group of Wen Lai has reported the study of the effect of the 129DISC1^Del^ mutation in pure B6 background (Juan et al., 2014). This model has modest behavioral phenotype, showing significant changes only in working memory. By contrast, the model here presented based also in the natural 25-bp deletion, shows alterations in spontaneous locomotion/activity, PPI and FST thus modelling more aspects of schizophrenia and other mental illnesses present in the Scottish family. The advantage presented by our model seems to rely on genetic background. In our case, behavioral consequences of DISC1 truncation have been studied in a mixed B6129F2 background. General practice try to avoid the use of mixed background due to concerns relating to chance of segregation or close linkage of modifier genes or also to sample size. Unlike these theoretical limitations, there are studies supporting the validity of using mixed background in behavioral studies (Estill et al., 2001). Moreover, some studies point that inbred strains show no advantage regarding variability in comparison to mixed strains (Estill et al., 2001). In the case of using an inbred background one has to be cautious when choosing the mouse strain. Namely, for despair behavior, it seems that the B6 strain presents high immobility time in both FST and TST (Jacobson and Cryan, 2007), thus making difficult to see a subtle increase due to a genetic alteration. Interestingly, 129 strains seem to spend less time immobile when compared to B6. Also, there is evidence that B6 strain displays a weaker response to contextual and cued fear conditioning and low PPI, which makes it less sensitive to a decrease caused by gene modifications (Paylor and Crawley, 1997; Bothe et al., 2004). In the present study we used mixed background B6129F2 which resulted to be more sensitive to increases in immobility time in FST and TST and to decreases in PPI and possible alterations in cued fear conditioning. For the future we plan to study the effects of DISC1 deficiency in 129 background compared to 129 free of 25-bp deletion.

Overall, our +/del model based on a naturally occurring mutation of DISC1, represents a mouse model that encompasses several phenotypes related to schizophrenia and other mental illnesses diagnosed in the Scottish family. Besides, as it is based in the use of a widely and commercially available mouse strain, it can become an amenable tool to study psychiatric behaviors.

## Conflict of interest statement

The authors declare that the research was conducted in the absence of any commercial or financial relationships that could be construed as a potential conflict of interest.

## References

[B1] Arguello P. A. Gogos J. A. (2006). Modeling madness in mice: one piece at a time. Neuron 52, 179–196 10.1016/j.neuron.2006.09.023 17015235

[B2] Blackwood D. H. Fordyce A. Walker M. T. St Clair D. M. Porteous D. J. Muir W. J. (2001). Schizophrenia and affective disorders—cosegregation with a translocation at chromosome 1q42 that directly disrupts brain-expressed genes: clinical and P300 findings in a family. Am. J. Hum. Genet. 69, 428–433 10.1086/321969 11443544 PMC1235314

[B3] Bothe G. W. Bolivar V. J. Vedder M. J. Geistfeld J. G. (2004). Genetic and behavioral differences among five inbred mouse strains commonly used in the production of transgenic and knockout mice. Genes Brain Behav. 3, 149–157 10.1111/j.1601-183x.2004.00064.x 15140010

[B4] Brandon N. J. Sawa A. (2011). Linking neurodevelopmental and synaptic theories of mental illness through DISC1. Nat. Rev. Neurosci. 12, 707–722 10.1038/nrn3120 22095064 PMC3954824

[B5] Cash-Padgett T. Jaaro-Peled H. (2013). DISC1 mouse models as a tool to decipher gene-environment interactions in psychiatric disorders. Front. Behav. Neurosci. 7:113 10.3389/fnbeh.2013.00113 24027503 PMC3759735

[B6] Castagné V. Moser P. Roux S. Porsolt R. D. (2011). Rodent models of depression: forced swim and tail suspension behavioral despair tests in rats and mice. Curr. Protoc. Neurosci. Chapter 8:Unit 8.10A 10.1002/0471141755.ph0508s38 21462162

[B7] Castagné V. Moser P. C. Porsolt R. D. (2009a). Preclinical behavioral models for predicting antipsychotic activity. Adv. Pharmacol. 57, 381–418 10.1016/s1054-3589(08)57010-4 20230767

[B8] Castagné V. Porsolt R. D. Moser P. (2009b). Use of latency to immobility improves detection of antidepressant-like activity in the behavioral despair test in the mouse. Eur. J. Pharmacol. 616, 128–133 10.1016/j.ejphar.2009.06.018 19549518

[B9] Clapcote S. J. Roder J. C. (2006). Deletion polymorphism of Disc1 is common to all 129 mouse substrains: implications for gene-targeting studies of brain function. Genetics 173, 2407–2410 10.1534/genetics.106.060749 16751659 PMC1569715

[B10] Clapcote S. J. Lipina T. V. Millar J. K. Mackie S. Christie S. Ogawa F. (2007). Behavioral phenotypes of Disc1 missense mutations in mice. Neuron 54, 387–402 10.1016/j.neuron.2007.04.015 17481393

[B11] Cryan J. F. Holmes A. (2005). The ascent of mouse: advances in modelling human depression and anxiety. Nat. Rev. Drug Discov. 4, 775–790 10.1038/nrd1825 16138108

[B12] Cryan J. F. Mombereau C. Vassout A. (2005). The tail suspension test as a model for assessing antidepressant activity: review of pharmacological and genetic studies in mice. Neurosci. Biobehav. Rev. 29, 571–625 10.1016/j.neubiorev.2005.03.009 15890404

[B13] Estill S. J. Fay K. Garcia J. A. (2001). Statistical parameters in behavioral tasks and implications for sample size of C57BL/6J:129S6/SvEvTac mixed strain mice. Transgenic Res. 10, 157–175 10.1023/A:1008955016170 11305362

[B14] Goel N. Bale T. L. (2009). Examining the intersection of sex and stress in modelling neuropsychiatric disorders. J. Neuroendocrinol. 21, 415–420 10.1111/j.1365-2826.2009.01843.x 19187468 PMC2716060

[B15] Gogos J. A. Gerber D. J. (2006). Schizophrenia susceptibility genes: emergence of positional candidates and future directions. Trends Pharmacol. Sci. 27, 226–233 10.1016/j.tips.2006.02.005 16530856

[B16] Gould T. D. Dao D. T. (2009). “The open field test,” in Mood and Anxiety Related Phenotypes in Mice: Characterization using Behavioral Tests, ed Gould T. D. (New York: Humana Press), 1–20

[B17] Hikida T. Jaaro-Peled H. Seshadri S. Oishi K. Hookway C. Kong S. (2007). Dominant-negative DISC1 transgenic mice display schizophrenia-associated phenotypes detected by measures translatable to humans. Proc. Natl. Acad. Sci. U S A 104, 14501–14506 10.1073/pnas.0704774104 17675407 PMC1964873

[B18] Jaaro-Peled H. (2009). Gene models of schizophrenia: DISC1 mouse models. Prog. Brain Res. 179, 75–86 10.1016/S0079-6123(09)17909-8 20302820

[B19] Jacobson L. H. Cryan J. F. (2007). Feeling strained? Influence of genetic background on depression-related behavior in mice: a review. Behav. Genet. 37, 171–213 10.1007/s10519-006-9106-3 17029009

[B20] Jope R. S. Roh M. S. (2006). Glycogen synthase kinase-3 (GSK3) in psychiatric diseases and therapeutic interventions. Curr. Drug Targets 7, 1421–1434 10.2174/1389450110607011421 17100582 PMC1850891

[B21] Juan L. W. Liao C. C. Lai W. S. Chang C. Y. Pei J. C. Wong W. R. (2014). Phenotypic characterization of C57BL/6J mice carrying the Disc1 gene from the 129S6/SvEv strain. Brain Struct. Funct. 219, 1417–1431 10.1007/s00429-013-0577-8 23689501

[B22] Kato T. (2008). Molecular neurobiology of bipolar disorder: a disease of ‘mood-stabilizing neurons’? Trends Neurosci. 31, 495–503 10.1016/j.tins.2008.07.007 18774185

[B23] Kellendonk C. Simpson E. H. Kandel E. R. (2009). Modeling cognitive endophenotypes of schizophrenia in mice. Trends Neurosci. 32, 347–358 10.1016/j.tins.2009.02.003 19409625 PMC4928481

[B24] Kohl S. Heekeren K. Klosterkotter J. Kuhn J. (2013). Prepulse inhibition in psychiatric disorders—apart from schizophrenia. J. Psychiatr. Res. 47, 445–452 10.1016/j.jpsychires.2012.11.018 23287742

[B25] Koike H. Arguello P. A. Kvajo M. Karayiorgou M. Gogos J. A. (2006). Disc1 is mutated in the 129S6/SvEv strain and modulates working memory in mice. Proc. Natl. Acad. Sci. U S A 103, 3693–3697 10.1073/pnas.0511189103 16484369 PMC1450143

[B26] Kuroda K. Yamada S. Tanaka M. Iizuka M. Yano H. Mori D. (2011). Behavioral alterations associated with targeted disruption of exons 2 and 3 of the Disc1 gene in the mouse. Hum. Mol. Genet. 20, 4666–4683 10.1093/hmg/ddr400 21903668

[B27] Kvajo M. Mckellar H. Arguello P. A. Drew L. J. Moore H. Macdermott A. B. (2008). A mutation in mouse Disc1 that models a schizophrenia risk allele leads to specific alterations in neuronal architecture and cognition. Proc. Natl. Acad. Sci. U S A 105, 7076–7081 10.1073/pnas.0802615105 18458327 PMC2383956

[B28] Kvajo M. Mckellar H. Drew L. J. Lepagnol-Bestel A. M. Xiao L. Levy R. J. (2011). Altered axonal targeting and short-term plasticity in the hippocampus of Disc1 mutant mice. Proc. Natl. Acad. Sci. U S A 108, E1349–E1358 10.1073/pnas.1114113108 22049344 PMC3241761

[B29] Li W. Zhou Y. Jentsch J. D. Brown R. A. Tian X. Ehninger D. (2007). Specific developmental disruption of disrupted-in-schizophrenia-1 function results in schizophrenia-related phenotypes in mice. Proc. Natl. Acad. Sci. U S A 104, 18280–18285 10.1073/pnas.0706900104 17984054 PMC2084334

[B30] Ma T. M. Abazyan S. Abazyan B. Nomura J. Yang C. Seshadri S. (2013). Pathogenic disruption of DISC1-serine racemase binding elicits schizophrenia-like behavior via D-serine depletion. Mol. Psychiatry 18, 557–567 10.1038/.mp.2012.97 22801410 PMC3475769

[B31] Millar J. K. Wilson-Annan J. C. Anderson S. Christie S. Taylor M. S. Semple C. A. (2000). Disruption of two novel genes by a translocation co-segregating with schizophrenia. Hum. Mol. Genet. 9, 1415–1423 10.1093/.hmg/.9.9.1415 10814723

[B32] Mukai J. Liu H. Burt R. A. Swor D. E. Lai W. S. Karayiorgou M. (2004). Evidence that the gene encoding ZDHHC8 contributes to the risk of schizophrenia. Nat. Genet. 36, 725–731 10.1038/ng1375 15184899

[B33] Paterlini M. Zakharenko S. S. Lai W. S. Qin J. Zhang H. Mukai J. (2005). Transcriptional and behavioral interaction between 22q11.2 orthologs modulates schizophrenia-related phenotypes in mice. Nat. Neurosci. 8, 1586–1594 10.1038/nn1562 16234811

[B34] Paylor R. Crawley J. N. (1997). Inbred strain differences in prepulse inhibition of the mouse startle response. Psychopharmacology (Berl) 132, 169–180 10.1007/s002130050333 9266614

[B35] Petit-Demouliere B. Chenu F. Bourin M. (2005). Forced swimming test in mice: a review of antidepressant activity. Psychopharmacology (Berl) 177, 245–255 10.1007/s00213-004-2048-7 15609067

[B36] Picchioni M. M. Murray R. M. (2007). Schizophrenia. BMJ 335, 91–95 10.1136/bmj.39227.616447.BE 17626963 PMC1914490

[B37] Piccinelli M. Wilkinson G. (2000). Gender differences in depression. Critical review. Br. J. Psychiatry 177, 486–492 10.1192/bjp.177.6.486 11102321

[B38] Pletnikov M. V. (2009). Inducible and conditional transgenic mouse models of schizophrenia. Prog. Brain Res. 179, 35–47 10.1016/s0079-6123(09)17905-0 20302816

[B39] Pletnikov M. V. Ayhan Y. Nikolskaia O. Xu Y. Ovanesov M. V. Huang H. (2008). Inducible expression of mutant human DISC1 in mice is associated with brain and behavioral abnormalities reminiscent of schizophrenia. Mol. Psychiatry 13, 173–186 10.1038/sj.mp.4002079 17848917

[B40] Porsolt R. D. Anton G. Blavet N. Jalfre M. (1978). Behavioural despair in rats: a new model sensitive to antidepressant treatments. Eur. J. Pharmacol. 47, 379–391 10.1016/0014-2999(78)90118-8 204499

[B41] Powell S. B. Zhou X. Geyer M. A. (2009). Prepulse inhibition and genetic mouse models of schizophrenia. Behav. Brain Res. 204, 282–294 10.1016/j.bbr.2009.04.021 19397931 PMC2735602

[B42] Ross C. A. Margolis R. L. Reading S. A. Pletnikov M. Coyle J. T. (2006). Neurobiology of schizophrenia. Neuron 52, 139–153 10.1016/j.neuron.2006.09.015 17015232

[B43] Shen S. Lang B. Nakamoto C. Zhang F. Pu J. Kuan S. L. (2008). Schizophrenia-related neural and behavioral phenotypes in transgenic mice expressing truncated Disc1. J. Neurosci. 28, 10893–10904 10.1523/JNEUROSCI.3299-08.2008 18945897 PMC6671369

[B44] Stark K. L. Xu B. Bagchi A. Lai W. S. Liu H. Hsu R. (2008). Altered brain microRNA biogenesis contributes to phenotypic deficits in a 22q11-deletion mouse model. Nat. Genet. 40, 751–760 10.1038/ng.138 18469815

[B45] Wexler E. M. Geschwind D. H. (2011). DISC1: a schizophrenia gene with multiple personalities. Neuron 72, 501–503 10.1016/j.neuron.2011.10.023 22099453

